# Highly Efficient Generation of Pigs Harboring a Partial Deletion of the CD163 SRCR5 Domain, Which Are Fully Resistant to Porcine Reproductive and Respiratory Syndrome Virus 2 Infection

**DOI:** 10.3389/fimmu.2019.01846

**Published:** 2019-08-08

**Authors:** Chunhe Guo, Min Wang, Zhenbang Zhu, Sheng He, Hongbo Liu, Xiaofeng Liu, Xuan Shi, Tao Tang, Piao Yu, Jianhua Zeng, Linfang Yang, Yongchang Cao, Yaosheng Chen, Xiaohong Liu, Zuyong He

**Affiliations:** ^1^State Key Laboratory of Biocontrol, Guangzhou Higher Education Mega Center, School of Life Sciences, Sun Yat-sen University, Guangzhou, China; ^2^Guangdong YIHAO Food Co., Ltd., Guangzhou, China

**Keywords:** PRRSV, CD163, SRCR5, CRISPR/Cas9, resistance

## Abstract

Porcine reproductive and respiratory syndrome virus (PRRSV) 1 and 2 differ in their recognition of CD163. Substitution of porcine CD163 SRCR5 domain with a human CD163-like SRCR8 confers resistance to PRRSV 1 but not PRRSV 2. The deletion of CD163 SRCR5 has been shown to confer resistance to PRRSV 1 *in vivo* and both PRRSV 1 and 2 *in vitro*. However, the anti-PRRSV 2 activity of modifying the CD163 SRCR5 domain has not yet been reported. Here, we describe the highly efficient generation of two pig breeds (Liang Guang Small Spotted and Large White pigs) lacking a short region of CD163 SRCR5, including the ligand-binding pocket. We generated a large number of gene-edited Large White pigs of the F0 generation for use in viral challenge studies. The results of this study show that these pigs are completely resistant to infection by species 2 PRRSV, JXA1, and MY strains. There were no clinical symptoms, pathological abnormalities, viremia, or anti-PRRSV antibodies in the CD163 SRCR5-edited pigs compared to wild-type controls after viral challenge. Porcine alveolar macrophages (PAMs) isolated from CD163 SRCR5-edited Large White pigs also displayed resistance to PRRSV *in vitro*. In addition, CD163 SRCR5-edited PAMs still exhibited a cytokine response to PRRSV infection, and no significant difference was observed in cytokine expression compared to wild-type PAMs. Taken together, these data suggest that CD163 SRCR5-edited pigs are resistant to PRRSV 2, providing a basis for the establishment of PRRSV-resistant pig lines for commercial application and further investigation of the essential region of SRCR5 involved in virus infection.

## Introduction

Porcine reproductive and respiratory syndrome (PRRS), caused by PRRS virus (PRRSV), is one of the most prevalent and serious infectious diseases in the global swine industry (1). Since it was first described in 1987 in the United States, PRRS has caused huge economic losses worldwide, especially in China (2–5). Pigs infected with PRRSV generally present with symptoms including fever, depression, and weight loss. PRRS is characterized by reproductive disorders involving abortion, stillbirth, weak piglets in pregnant sows and severe respiratory symptoms in piglets (6, 7). The high morbidity and mortality of PRRSV infection seriously affect the development of the swine industry, making research on PRRSV a constant focus.

PRRSV is a single-stranded, positive-sense RNA virus and is classified into species 1 PRRSV (European species) and species 2 PRRSV (North American species) (8). The genome of PRRSV is about 15 kb in length and contains at least 11 open reading frames (ORFs). PRRSV exhibits tropism for specific subsets of the monocyte/macrophage lineage and primarily replicates in porcine alveolar macrophages (PAMs) (9). The infection of PRRSV on host cells depends on receptor-mediated adsorptive endocytosis (10). Cellular molecules including heparan sulfate, CD163, sialoadhesin (CD169), CD151, vimentin, and DC-SIGN (CD209) have been described as potential receptors for PRRSV (11, 12). Among these cellular receptors, CD163 has been identified as the essential receptor mediating both species 1 and 2 PRRSV infection (13–16). However, PRRSV species 1 and 2 differ in their recognition of CD163 (17).

CD163 is a member of the scavenger receptor cysteine-rich (SRCR) family, expressed on the cell surface and in early endosomes of PAMs. The CD163 protein is comprised of nine SRCR domains, a transmembrane segment and a cytoplasmic tail (13, 18). It has been proven to interact with PRRSV GP2a and GP4 (19), facilitating the uncoating and release of the viral genome to the cytoplasm at a low pH within the early endosome (20). Compared with the other eight SRCR domains, the deletion of fifth SRCR domain (SRCR5) encoded by exon 7 appears to inhibit PRRSV infection, indicating that SRCR5 plays an important role in this process (21, 22). Two regions of SRCR5 have been reported to be involved in PRRSV infection (13): loop 5–6 (23), and the ligand-binding pocket (LBP) (24). Multiple binding sites on the outside of CD163 are important for its interaction with PRRSV. The first CD163 knockout pigs were born in 2016 and are resistant to the infection of species 2 PRRSV isolate (25, 26). In 2018, CD163 knock-out Duroc pigs were generated in China that are resistant to Chinese highly pathogenic PRRSV (HP-PRRSV) (27). Since CD163 is important for a variety of biological functions, the complete knockout of this gene could have a negative physiological impact on the animal. To maintain the biological functions of CD163, one study has substituted the porcine CD163 SRCR5 with a human CD163-like SRCR8, which confers resistance of pigs to PRRSV 1 but not PRRSV 2 (17). Recently, studies have shown that deletion of the entire CD163 SRCR5 could confer resistance to PRRSV 1 *in vivo* and both PRRSV 1 and 2 *in vitro* while maintaining the biological function of CD163 (28). However, whether a more precise modification of CD163 that has the ability to confer resistance of pigs to PRRSV 2 has not yet been reported.

In this study, we precisely deleted a 41-aa fragment containing the LBP in the SRCR5 domain of CD163 in two pig breeds (Liang Guang Small Spotted and Large White pigs). Gene-edited Large White pigs in the F0 generation were then used for viral challenge. These gene edited pigs and their respective PAMs were resistant to PRRSV 2 infection. Furthermore, we also investigated other biological functions of both membranous and soluble CD163 in order to determine whether its normal physiological functions were altered after CD163 gene editing.

## Materials and Methods

### Vector Construction

**Figure 1:**
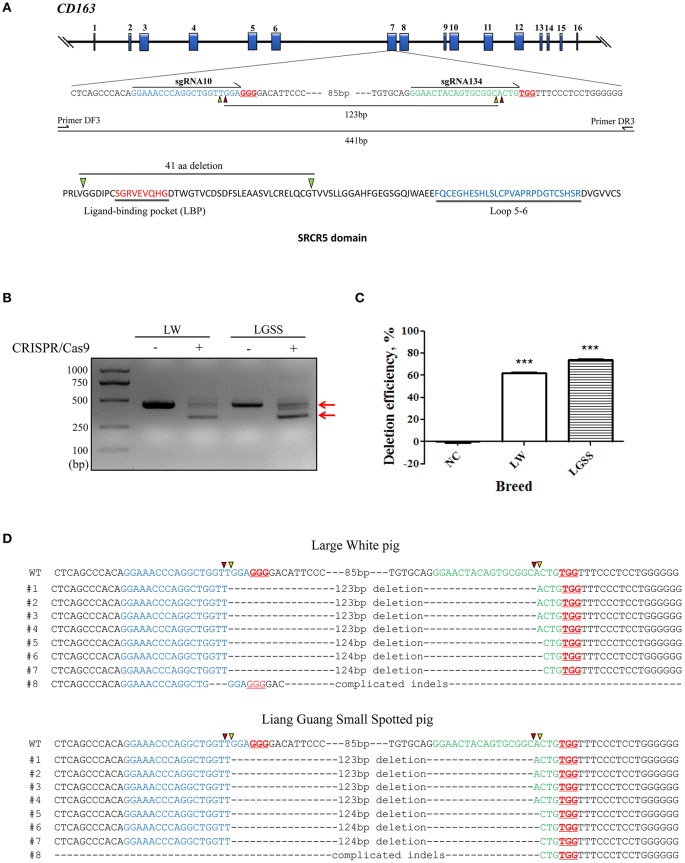
Generation of the precise partial deletion of CD163 SRCR5 in porcine embryonic fibroblasts (PEFs) using CRISPR/Cas9. **(A)** Schematic of the CD163 gene and target sites of sgRNAs designed for targeting SRCR5 in the exon 7. The 16 exons of CD163 are indicated by blue rectangles. Arrows indicate the sequence used for the guide segment of sgRNA10 and 134. The NGG nucleotide protospacer adjacent motif sequences are underlined in red. Red and yellow triangles represent the predicted cleavage sites of sgRNAs. A precise excision with paired sgRNAs results in a 123 bp in-frame deletion including ligand-binding pocket (LBP). The primer pair DF3/DR3 was used to amplify a 441 bp product from the intact allele of CD163 gene and a truncated product of 317 bp if the deletion (123 bp) has occurred. Two regions (LBP and loop 5–6) of SRCR5 are shown. **(B)** PCR products identifying the presence of the targeted deletion of CD163 SRCR5 induced by paired sgRNAs. The upper red arrow indicates the position of the 441 bp full length PCR product, and the lower red arrow indicates the expected positions of the truncated PCR product in the event of deletion. LW, Large White pig; LGSS, Liang Guang Small Spotted pig; M, marker. **(C)** The efficiency of the targeted deletion in PEFs was quantified by qPCR. ^***^p < 0.001 compared to negative control. **(D)** Sequence analysis of cloned PCR products. The guide segments of sgRNA 10 and 134 are shown in blue and green, respectively. Red and yellow triangles represent the predicted cleavage sites of sgRNAs. WT, wild-type DNA sequence. Data are representative of the results of three independent experiments (means ± SE). Significant differences are indicated as follows: ^***^p < 0.001.

The two sgRNAs, designated as CRISPR 10 and CRISPR 134, used for the deletion of nearly half of exon 7 of the porcine CD163 gene (Figure 1A) were selected from a previous study (29). Oligos of each sgRNA were cloned downstream of the human U6 promoter through *Bbs* I restriction sites in plasmid pSpCas9 (BB)-2A-GFP (pX458) (Addgene plasmid #48138) and our previously constructed plasmid pSpCas9(BB)-2A-DsRed (pX458R) (30) to create plasmids pX458-CRISPR 10 and pX458R-CRISPR 134. The positive clones were confirmed by Sanger sequencing (Sangon Biotech, China).

### Porcine Embryonic Fibroblast Culture and Transfection

Porcine embryonic fibroblasts (PEFs) were isolated from 35-day-old embryos. Briefly, the back tissue of the embryos was separated, then cut into pieces of 1 mm^3^ with scissors. The pieces were then placed in dishes filled with Dulbecco's modified Eagle's medium (DMEM) (Corning, USA) containing L-glutamine and 1 g/L D-glucose, supplemented with 20% fetal bovine serum (FBS) (PAN, Germany), 100 units/mL penicillin and 100 μg/ml streptomycin (Sigma, USA). The dishes were then placed in a humidified 37°C tissue culture incubator with 5% CO_2_ (Thermo, USA). After 3 days in culture, PEFs were harvested. For transfection, PEFs were resuspended in 100 μL buffer R (Invitrogen, USA), which contained 5 μg plasmid pX458-CRISPR 10 and 5 μg plasmid pX458R-CRISPR 134. The mixture was then transfected through electroporation at 1,650 V for 10 ms in 3 pulses using the Neon transfection system (Invitrogen, USA).

### Assessment of the Efficiency of the Paired sgRNAs for Targeted Deletion

Genomic DNA of sorted dual fluorescent cells was extracted using the DNeasy Blood and Tissue Kit (Qiagen, Germany). A pair of primers (DF3: 5′-CTGCTCAGCCCACAGGAAAC-3′; DR3: 5′-GCCATTCACCAAGCGGATTT-3′) were designed for PCR across the target sites of the paired sgRNAs. The PCR yields a 441 bp product from the intact allele of CD163, and a truncated product of 317 bp will be amplified if the deletion (123 bp) has occurred (Figure 1A). The percentage of deletion events was quantified through a qPCR method as described previously (30).

### Somatic Cell Nuclear Transfer (SCNT) and Embryo Transfer

To produce cloned gene-edited embryos, oocytes isolated from ovaries collected from a local abattoir were matured *in vitro* as described previously (31). SCNT was manipulated as previously described (32). Briefly, the polar body along with a portion of the adjacent cytoplasm, presumably containing the metaphase II plate, was removed, and a donor cell with dual fluorescence was placed in the perivitelline space. Cloned gene-edited embryos were transferred into Large White sow recipients on day 1 after first standing estrus.

### Genotyping

Genomic DNA was extracted from ear biopsies taken from piglets using the DNeasy Blood and Tissue Kit (Qiagen, Germany). The primer pair DF3/DR3 was used for amplification. Both full length and truncated fragments were subsequently cloned into the pMD18-T vector (Takara, Japan) for Sanger sequencing.

### Animals

Animals were provided by Muyuan Foodstuff Co, Ltd, Henan, China. All animal experiments were approved by the Institutional Animal Care and Use Committee of Sun Yat-sen University of China and the ethics group at Muyuan Food stuff Co, Ltd. All animal work was carried out under the Laboratory Animals—Guideline of welfare and ethics written by the General Administration of Quality Supervision, Inspection and Quarantine of the People's Republic of China.

### Cell Culture and Virus Production

Porcine alveolar macrophages (PAMs) were isolated from CD163 SRCR5-edited piglets and WT piglets. The piglets were euthanized and the lungs were obtained from the thoracic cavity. Sterile phosphate buffer solution (PBS, Corning, USA) was poured into the lungs from the trachea three times. Lungs were kneaded gently for about 15 min after lung each lavage in order to shed alveolar macrophages from the alveolar wall. Bronchoalveolar lavage fluid (BALF) was recycled. Cells were resuspended in 40% RPMI-1640 medium (Gibco, USA), 50% fetal bovine serum, and 10% DMSO (Sigma, USA), and stored in liquid nitrogen. No antibiotic was used in the culture during the experiments.

PRRSV JXA1 strain (a species 2 PRRSV isolate) was provided by Dr. Heng Wang from South China Agricultural University. PRRSV MY strain (also a species 2 PRRSV isolate) was isolated from Muyuan Foodstuff Co, Ltd., Henan, China. These two virus strains were propagated in Marc-145 cells and titrated to 50% tissue culture infective dose (TCID_50_).

### Challenge With PRRSV 2 Strains JXA1 and MY

Eight CD163 SRCR5-edited Large White piglets (genotypes shown in Table S3) and eight WT piglets at age 4–6 weeks that were negative for the PRRSV antigen and antibody were selected for viral challenge. These piglets were divided into two groups to challenge with two different PRRSV strains. Four CD163 SRCR5-edited piglets and four WT piglets were set up as a group. These eight piglets were kept at a suitable temperature and humidity level, and water and food were available *ad libitum*. After 1 week of acclimation, piglets were challenged with PRRSV using a nasal drip. One group was injected with PRRSV JXA1 strain, the other group was challenged with the MY strain. The dose of intranasal injection was 2 × 10^5^ TCID_50_ JXA1 or MY diluted in 3 mL culture medium. Blood samples were collected on days 0, 7, 14, 21, 28, 35, and 42 post challenge for the detection of PRRSV antigen and antibody. Rectal temperature and weight were measured on the aforementioned days. In addition, clinical symptoms including respiratory symptoms and neurological signs were recorded and scored every day post challenge. Any animal deaths during the PRRSV challenge were recorded and the mortality was calculated. On day 42, all animals were euthanized.

### Hematoxylin and Eosin (H and E) Staining and Immunohistochemistry

All animals were euthanized on day 42 of the PRRSV challenge. Lungs were isolated from euthanized WT pigs and gene-edited pigs. Pictures were taken of the dorsal side of the lung and the lungs were evaluated for histopathology. Lung samples were fixed in 10% neutral buffered formalin (Ruishu, China). Fixed sections were embedded in paraffin and 5 μm thick sections were cut and mounted to glass slides. For histopathology, sections were stained with H&E (NJJCTECH, China) according to the manufacturer's instructions. Immunohistochemistry was performed with the Cell and Tissue Staining Kit (R&D, USA) to detect the PRRSV antigen. The primary antibody used was monoclonal anti-PRRSV N protein (1:1,000 dilution, Jeno Biotech, Inc., Republic of Korea). Images were acquired using a fluorescence microscope (NIKON ECLIPSE Ti2-U, Japan).

### Detection of Viral Copy Number and Anti-PRRSV Antibody Levels in Serum

Serum samples were collected on days 0, 7, 14, 21, 28, 35, and 42 post PRRSV challenge. To detect the PRRSV antigen in serum, qRT-PCR was used. Viral RNA was extracted using RaPure Viral RNA/DNA Kit (Magen, China) and quantified by qRT-PCR using VetMAX™ PRRSV NA and EU Reagents (Thermo Fisher Scientific, USA). Ct values were calculated for absolute PRRSV RNA quantity (copy number) according to the standard curve produced by the different dilutions of the positive PRRSV RNA control. IDEXX PRRS X3 Ab (IDEXX Laboratories Inc., USA) was used to detect the anti-PRRSV antibody in accordance with the manufacturer's instructions. S/P reflected the level of antibody. Results were reported as negative (ELISA sample to positive [S/P] ratio of <0.4) or positive (ELISA S/P ratio of ≥0.4).

### Measurement of Soluble CD163 in Cell Supernatants

CD163 SRCR5-edited PAMs and WT PAMs were seeded in six-well plates and cultured for 24 h. The supernatants were collected for the detection of soluble CD163. A porcine CD163 ELISA kit (Laibio, China) was used to measure absorbance (450 nm) according to the manufacturer's instructions.

### Flow Cytometry

Cells were harvested and fixed with 4% paraformaldehyde (Ruishu, China) for 10 min. After rinsing with PBS three times, they were incubated with an anti-pig CD163-FITC antibody (1:500 dilution, Bio-Rad) for 1 h at room temperature. Finally, cells were washed with PBS three times and resuspended in PBS at a concentration of 1 × 10^6^ cells/ml. Flow cytometry was used to analyze the ratio of FITC-positive cells. Approximately 10,000 labeled cells were counted using a FACSCalibur (BD Bioscience, USA) and analyzed by FlowJo software.

### Quantitative Real-Time Reverse-Transcription Polymerase Chain Reaction (qRT-PCR)

Total RNA was extracted from cultured cells using TRIzol (Magen, China). The Reverse Transcription System (Promega, USA) was used to reverse transcribe 1 μg of total RNA to cDNA. Relative expression of target genes was calculated using the 2^−ΔΔ*Ct*^ method and normalized to the mean Ct of GAPDH. The primers used for qRT-PCR are listed in Table S4.

### Analysis of Cytokine Levels in PAMs

CD163 SRCR5-edited and WT PAMs were seeded in six-well plates. When adhered to the plates, cells were infected with JXA1 (MOI = 1) for 24 h. Total RNA was extracted from cultured cells using TRIzol (Magen, China). After reverse transcription, the expression of cytokines (IL-1β, IL-8, IL-10, and IFN-α) was detected quantitatively via qRT-PCR. The primers used for qRT-PCR are listed in Table S4.

### Statistical Analysis

All experiments were performed with at least three independent replicates. Student's *t*-test and one-way ANOVA were used to analyze the data. Statistical analysis was performed using SPSS 17.0 and GraphPad Prism 5.0. *P* < 0.05 was considered to be significant.

## Results

### Highly Efficient Deletion of a CD163 SRCR5 Fragment in Porcine Embryonic Fibroblasts by CRISPR/Cas9

To evaluate the efficiency of the targeted deletion within the CD163 SRCR5 domain, PEFs derived from both the Large White pig and the Liang Guang Small Spotted pig (Chinese indigenous breed) were transfected with pX458-CRISPR 10 (expressing an EGFP reporter) and pX458R-CRISPR 134 (expressing a DsRed reporter; Figure S1). Subsequently, the PEFs were sorted to obtain dual fluorescent cells and PCR was performed to amplify the region across the target sites of the paired sgRNAs (Figure 1A). The truncated PCR product was more abundant than the full-length product in PEFs from both pig breeds (Figure 1B), indicating efficient targeted deletion has occurred in the sorted cells. This was confirmed by quantifying the percentage of deletion events by qPCR analysis, which demonstrated 62% deletion efficiency in PEFs from the Large White pig and 74% in PEFs from the Liang Guang Small Spotted pig (Figure 1C).

Sequencing of the cloned truncated PCR products revealed that in most cases, the breakpoint junctions (sites that are three or four base pairs upstream of the protospacer adjacent motif induced by the pair of sgRNAs) were precisely rejoined (Figure 1D). This is consistent with our previous findings (30). Interestingly, in PEFs from both pig breeds, nearly half of the edited events resulted in a 123 bp in frame deletion within the SRCR5 domain of CD163, providing a strong basis for the generation of pigs with a deleted fragment of CD163 SRCR5.

### Efficient Generation of Pigs With a Small Deletion in CD163 SRCR5

**Figure 2:**
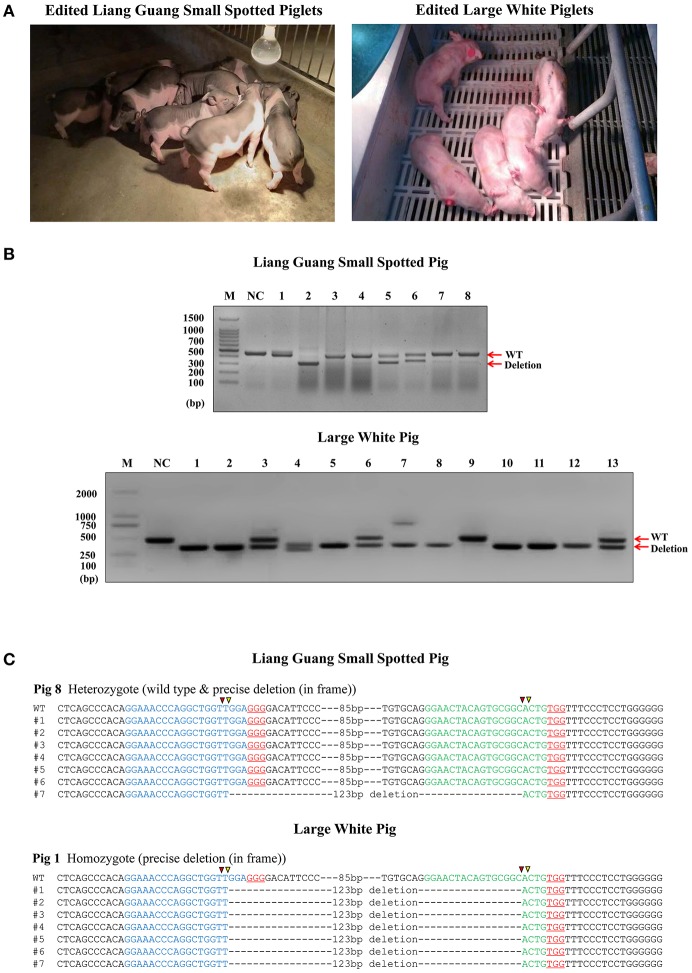
Generation of pigs harboring a precise partial deletion of CD163 SRCR5. **(A)** Representative photos of CD163-edited Liang Guang Small Spotted piglets and Large White piglets. **(B)** Genotyping of edited piglets. DNA was extracted from ear biopsies and genotype was assessed by PCR across the target sites of the paired sgRNAs. The PCR product of the unmodified genome is predicted to be 441 bp, while the deletion (123 bp) should result in a 317 bp PCR product. NC, negative control using the PCR product from wild-type genomic DNA. Each numbered lane indicates the PCR product from one healthy gene-edited piglet. **(C)** Sequencing of the cloned PCR products shows a representative Liang Guang Small Spotted piglet carrying a heterologous in frame deletion (123 bp) in CD163 SRCR5, and a representative Large White piglet carrying a homologous in frame deletion (123 bp) in CD163 SRCR5. Red and yellow triangles are predicted cutting sites of Cas9 nuclease.

Prior to embryo transfer, the *in vitro* developmental competency of cloned edited embryos was evaluated (Table S1), which showed that they are suitable for implantation into surrogate sows. Three Liang Guang Small Spotted pig and two Large White pig embryos were transferred into recipient sows (Table 1 and Figure S3). Seven Liang Guang Small Spotted pigs and eight Large White pigs of the recipient gilts carried their pregnancies to term, resulting in pregnancy rates of 64 and 80%, respectively. A total of 26 Liang Guang Small Spotted piglets and 46 Large White piglets were born alive, and a total of 8 Liang Guang Small Spotted piglets and 13 Large White piglets remained healthy during growth (Table 1 and Figure 2A).

**Table 1:** Summary of CD163 SRCR5-edited pigs generated through SCNT.

**Breed**	**No. of experiment**	**No. of transferred embryos**	**No. of recipients**	**No. (%) of pregnancies**	**Litter size**	**No. of born alive**	**No. of healthy piglets**	No. (%) of precise deletion^*^
LGSS	1	599	4	3 (75%)	7	5		
	2	304	2	1 (50%)	12	11		
	3	819	5	3 (60%)	10	10		
Total		1722	11	7 (64%)	29	26	8	2 (25%)
LW	1	1020	5	3 (60%)	16	10		
	2	1083	5	5 (100%)	46	36		
Total		2103	10	8 (80%)	62	46	13	11 (84.6%)

*LGSS, Liang Guang Small Spotted pig; LW, Large White pig*.

* *The percentage of precise deletion = No. of precise deletion/No. of healthy piglets*.

Genotyping of ear biopsies revealed that two of the eight (25%) healthy Liang Guang Small Spotted piglets had one deleted allele of CD163 SRCR5 (Figures 2B,C and Table S1, Figure S2), and 11 of 13 (85%) healthy Large White piglets had at least one deleted allele of CD163 SRCR5 (Figures 2B,C and Table S1, Figure S2).

Liang Guang Small Spotted piglet 7 and 8 were heterozygous for the in frame deletion (123 bp) of CD163 SRCR5. The gene was edited in the remaining six individuals, which presented diverse editing results (Table S2, Figure S2A). The 13 healthy Large White piglets were all edited at the CD163 locus. Piglet 1 and 8 both contained homologous in frame (123 bp) deletions of CD163 SRCR5 (Figure 2C and Figure S2B), and piglet 11 contained homologous out of frame deletions (124 bp) in CD163 SRCR5 (Figure S2B). The remaining individuals all presented with biallelic modifications (Table S3, Figure S2B).

### CD163 SRCR5-Edited Pigs Are Resistant to PRRSV Infection

**Figure 3:**
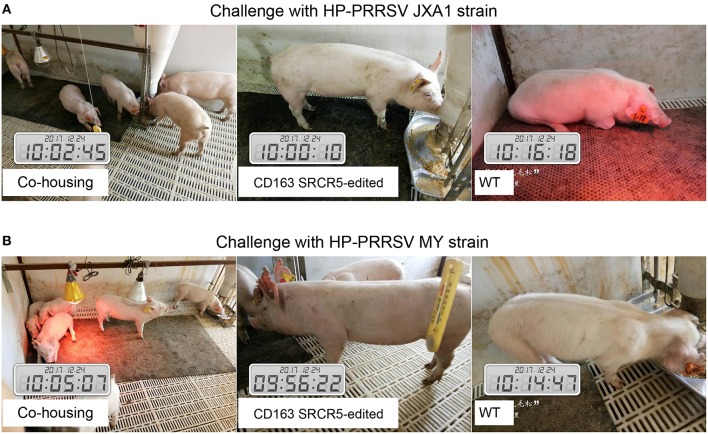
CD163 SRCR5-edited pigs do not show clinical symptoms after PRRSV challenge. **(A,B)** Sixteen piglets were divided into two groups, of which four CD163 SRCR5-edited Large White piglets and four WT piglets were mixed as a group. These piglets were co-housed and given access to food and water *ad libitum*. One group was challenged with the PRRSV JXA1 strain **(A)**, the other group was challenged with the PRRSV MY strain **(B)**. Clinical signs related to PRRSV, including respiratory and neurological symptoms, were observed and recorded every day post challenge. Pictures were taken on day 21 post challenge.

Sixteen piglets were divided into two groups for challenge with PRRSV JXA1 and MY strains. Four CD163 SRCR5-edited piglets and four WT piglets were co-housed. Symptoms including respiratory and neurological symptoms were observed and recorded daily post PRRSV challenge. A scoring system of 0–3 (33) was used to assess the clinical symptoms as follows: 0, normal; 1, mild dyspnea; 2, moderate dyspnea or tachypnea and inappetence; 3, severe tachypnea, anorexia, and depression. As shown in Figures 3A,B, CD163 SRCR5-edited piglets were generally healthy with normal food and water consumption after infection with PRRSV JXA1 and MY. However, virus-infected WT piglets displayed depression, anorexia, and drowsiness, in addition to respiratory problems and mucoid nasal discharge (average score, 3). These data suggest that CD163 SRCR5-edited piglets show no symptoms of PRRSV infection.

### CD163 SRCR5-Edited Pigs Are Histopathologically Normal After PRRSV Challenge

**Figure 4:**
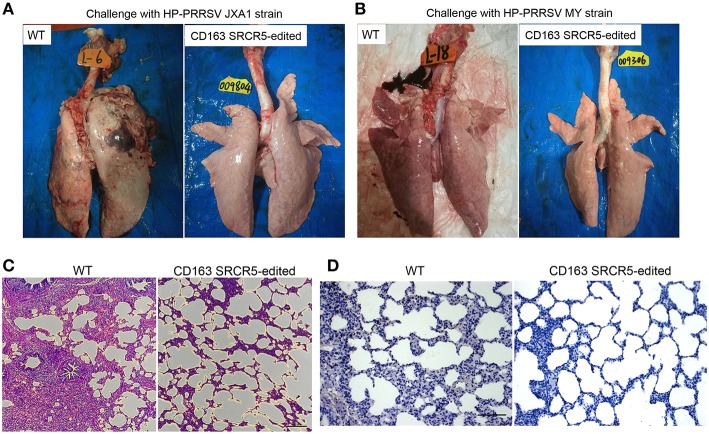
CD163 SRCR5-edited pigs exhibit normal histopathology after PRRSV challenge. Lungs were isolated from CD163 SRCR5-edited and WT animals on day 42 post PRRSV challenge. Pathological changes of lung lesions were observed and assessed using visual examination, Hematoxylin and Eosin (H&E) staining and immunohistochemistry. **(A,B)** Photographs of the dorsal side of lungs from CD163 SRCR5-edited and WT animals challenged with PRRSV JXA1 **(A)** and MY **(B)** strains. **(C)** Lung paraffin sections were stained with H&E (scale bar, 100 μm). **(D)** Immunohistochemistry analysis of the PRRSV antigen (brown) in lung paraffin sections (scale bar, 50 μm). The macrophages stain intensely dark brown due to the presence of the PRRSV antigen.

WT and CD163 SRCR5-edited animals were euthanized on day 42 post-PRRSV challenge. Lungs were isolated from the thoracic cavity and pathologically assessed using visual examination, H&E staining and immunohistochemistry. As shown in Figures 4A,B, lungs were photographed from the dorsal side. Severe hemorrhage, congestion and even necrosis occurred on the surface of the lungs from WT animals. However, petechiae were absent from the surface of the lungs from CD163 SRCR5-edited animals after challenge with JXA1 and MY strains. In order to assess the histopathological changes of WT and CD163 SRCR5-edited pigs, paraffin sections were stained with H&E. This analysis showed thickening of the alveolar walls and infiltration of a large number of inflammatory cells in the pulmonary interstitium in the lungs of WT pigs, suggesting the presence of diffuse interstitial pneumonia (Figure 4C). These pathological changes were not observed in the sections of CD163 SRCR5-edited pigs (Figure 4C). PRRSV antigens in the lung sections were detected using immunohistochemistry analysis. As shown in Figure 4D, the virus (brown) was detected in the lungs of WT animals, whereas PRRSV antigens were not present in the sections of CD163 SRCR5-edited animals post challenge with JXA1 and MY strains.

### CD163 SRCR5-Edited Pigs Survive After PRRSV Challenge

**Figure 5:**
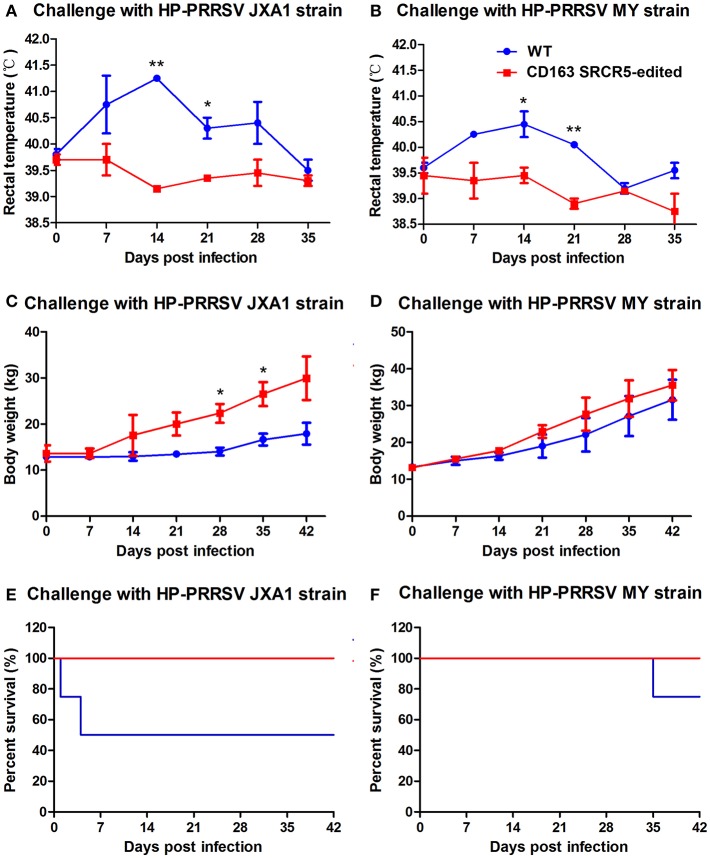
CD163 SRCR5-edited pigs survive post PRRSV challenge. **(A–F)** Rectal temperature **(A,B)** and body weights **(C,D)** were measured on day 0 prior to challenge and the days 7, 14, 21, 28, 35, 42 post-challenge with PRRSV JXA1 and MY strains. **(E,F)** The mortality and survival curve of piglets during PRRSV JXA1 **(E)** and MY **(F)** strain challenges. The red line represents CD163 SRCR5-edited pigs, and the blue line represents WT controls. Data are representative of the results of three independent experiments (means ± SE). Significant differences are indicated as follows: ^*^P < 0.05, ^**^P < 0.01.

Piglets were challenged with PRRSV JXA1 and MY strains using a nasal drip. Rectal temperatures and body weights of the animals were measured on day 0 prior to challenge and days 7, 14, 21, 28, 35, and 42 post-challenge. As shown in Figure 5A, the temperatures of WT piglets were remarkably higher than that of CD163 SRCR5-edited piglets. The temperatures of CD163 SRCR5-edited piglets remained in the normal range and varied between 38.5 and 39.5°C, while the temperatures of WT piglets were 0.5-1 degree higher and they developed a clinical fever, persisting over 40°C for ~28 days post JXA1 infection. The highest temperature of WT reached 40.5°C on day 14 post JXA1 infection (Figure 5B). Moreover, the body weights of CD163 SRCR5-edited piglets were higher compared to WT controls when challenged with PRRSV JXA1 and MY. The rate of weight gain of CD163 SRCR5-edited piglets was significantly higher than that of WT controls after day 7 post infection (Figures 5C,D). In addition, two of the JXA1-challenged WT pigs developed more severe symptoms and died within one week (Figure 5E), and one of MY-challenged WT pigs died on day 35 post infection (Figure 5F). However, CD163 SRCR5-edited piglets showed no signs or symptoms of infection and survived the PRRSV challenge.

### CD163 SRCR5-Edited Pigs Show No Viremia and Anti-PRRSV Antibody Response

**Figure 6:**
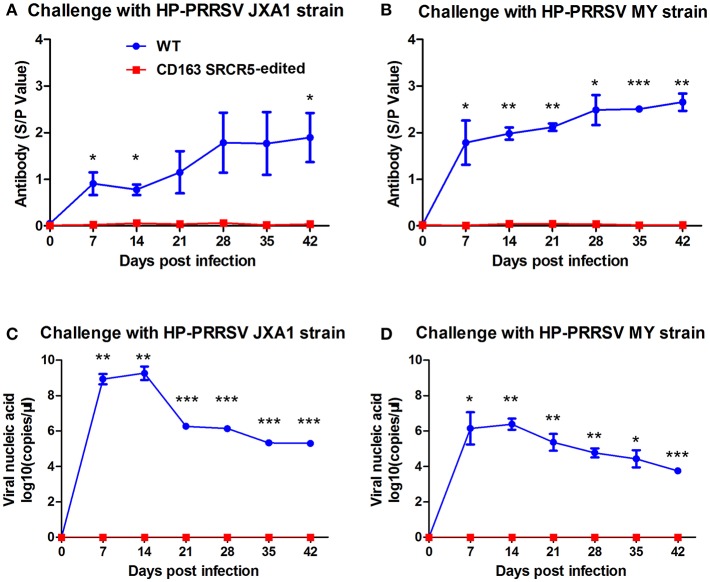
Viremia and anti-PRRSV antibodies are not present in CD163 SRCR5-edited animals. **(A,B)** Blood samples were collected to detect anti-PRRSV antibody titers on days 0, 7, 14, 21, 28, 35, and 42 post PRRSV JXA1 **(A)** and MY **(B)** challenge in CD163 SRCR5-edited animals and WT controls. The absorbance was detected at a wavelength of 570 nm. Antibody titers are represented as sample absorbance/positive absorbance (S/P). **(C,D)** Viral nucleic acid copy numbers in blood samples were measured on the designated days post JXA1 **(C)** and MY **(D)** inoculation of CD163 SRCR5-edited and WT animals. The red line represents CD163 SRCR5-edited pigs and the blue line represents WT controls. Data are representative of the results of three independent experiments (means ± SE). Significance is indicated as follows: ^*^P < 0.05, ^**^P < 0.01, ^***^P < 0.001.

In order to detect changes in PRRSV antigens and antibodies in WT and CD163 SRCR5-edited piglets, blood samples were collected on days 0, 7, 14, 21, 28, 35, and 42 post-challenge. Anti-PRRSV antibodies in WT piglets significantly increased during viral challenge, while the antibody levels of CD163 SRCR5-edited piglets remained negative (Figures 6A,B). Additionally, the levels of viral nucleic acids rapidly increased in WT piglets, but all CD163 SRCR5-edited piglets remained negative after challenge with two PRRSV strains (Figures 6C,D). These data demonstrate that CD163 SRCR5-edited piglets exhibit no PRRSV viremia and are resistant to PRRSV infection.

### Macrophages From CD163 SRCR5-Edited Pigs Are Resistant to PRRSV Infection *in vitro*

**Figure 7:**
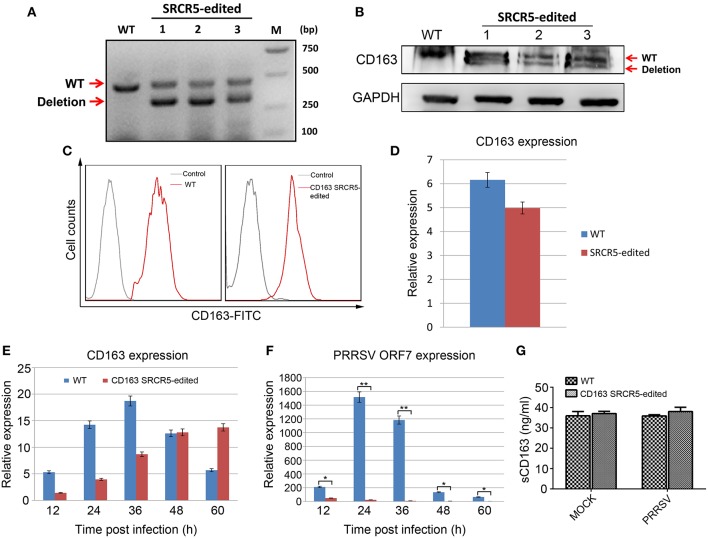
Resistance of PAMs isolated from CD163 SRCR5-edited piglets to PRRSV 2 *in vitro*. **(A)** RT-PCR analysis of CD163 expression in PAMs from three heterologous offspring of CD163 SRCR5-edited Large White pig 13. **(B)** Western blot analysis of CD163 protein expression from three heterologous offspring of CD163 SRCR5-edited Large White pig 13. **(C)** Expression of membranous CD163 in CD163 SRCR5-edited and WT PAMs was detected by flow cytometry. The gray line represents control cells and the red line represents the CD163-FITC positive cells. **(D)** CD163 mRNA expression was determined by qRT-PCR in SRCR5-edited cells and WT cells. **(E,F)** CD163 SRCR5-edited PAMs and WT controls were infected with JXA1 strain (MOI = 1). The expression of CD163 and PRRSV ORF7 was detected at 12, 24, 36, 48, and 60 h post infection (hpi). **(G)** CD163 SRCR5-edited PAMs and WT controls were either mock infected or infected with JXA1 strain (MOI = 1) for 24 h. The level of soluble CD163 in the supernatants was measured using an ELISA kit. Data are representative of the results of three independent experiments (means ± SE). Significant differences are indicated as follows: ^*^P < 0.05, ^**^P < 0.01.

Since PRRSV primarily replicates in PAMs, we sought to determine whether the PRRSV resistance of gene-edited pigs originated from antiviral properties of PAMs. To this end, we isolated PAMs from CD163 SRCR5-edited pigs for molecular characterization and PRRSV challenge. First, we detected the expression of cell membrane-localized CD163 in PAMs from WT and gene-edited pigs. RT-PCR analysis of PAMs from three heterologous offspring of CD163 SRCR5-edited Large White pig 13 (Figure S2B) clearly showed targeted deletion of CD163 transcript (Figure 7A). Sequencing of the cDNA revealing the 123 bp in-frame deletion in SRCR5 (Figure S2B) (data not shown). Western blot analysis of the same samples revealed a truncated CD163 protein (Figure 7B). To further confirm whether truncated CD163 was present on the cell surface of PAMs, cells were incubated with an anti-pig CD163-FITC antibody and analyzed by flow cytometry. There was no significant difference in the expression of CD163 between CD163 SRCR5-edited and WT PAMs (Figure 7C). The basal level of CD163 mRNA expression of SRCR5-edited cells was comparable to that of WT cells (Figure 7D). These results indicate that the small deletion in SRCR5 does not affect the expression or membrane targeting of CD163. Next, PAMs isolated from both edited and WT pigs were infected with the JXA1 strain, then CD163 mRNA expression was analyzed at 12, 24, 36, 48, and 60 h post infection (hpi). The expression of CD163 in SRCR5-edited PAMs was comparable to that of WT controls (Figure 7E). We also analyzed the expression of PRRSV ORF7, which indicates the occurrence of active replication of PRRSV. The expression of PRRSV ORF7 in CD163 SRCR5-edited PAMs was significantly lower than that of WT controls at all time points post infection, and PRRSV ORF7 was not expressed in CD163 SRCR5-edited PAMs at 24, 36, 48, and 60 hpi (Figure 7F). To determine whether soluble CD163 (sCD163) was affected, CD163 SRCR5-edited PAMs and WT PAMs were either mock infected or infected with JXA1 strain, and sCD163 in cell supernatants was assessed using a porcine CD163 ELISA kit. As shown in Figure 7G, sCD163 levels were found to be equal in supernatants of mock infected CD163 SRCR5-edited PAMs and WT PAMs, and were not significantly different between virus infected PRRSV-infected WT PAMs and CD163 SRCR5-edited PAMs. Taken together, these results suggest that PAMs from CD163 SRCR5-edited pigs are completely resistant to PRRSV infection. Moreover, modified CD163 exhibits normal biological function as a result of normal expression and localization of CD163 and sCD163.

### CD163 SRCR5-Edited PAMs Show a Cytokine Response to PRRSV Infection

**Figure 8:**
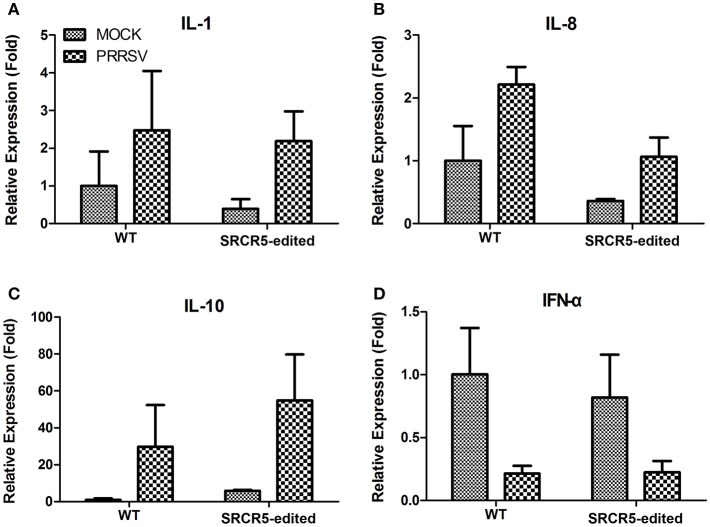
Expression of cytokines in PAMs isolated from CD163 SRCR5-edited piglets. **(A–D)** CD163 SRCR5-edited PAMs and WT PAMs were either mock infected or infected with JXA1 strain (MOI = 1) for 24 h. The expression of inflammatory cytokines IL-1β **(A)**, IL-8 **(B)**, IL-10 **(C)**, and IFN-α **(D)** were analyzed using qRT-PCR. Relative expression (fold) in comparison with mock infected WT PAMs (set up as 1) is shown. Data are representative of the results of three independent experiments (means ± SE).

To determine whether the biological function of modified cells is preserved with an intact immune response to PRRSV infection, PAMs isolated from CD163 SRCR5-edited and WT pigs were either mock infected or infected with JXA1 strain for 24 h. We conducted qRT-PCR to evaluate the expression of IL-1β, IL-8, IL-10, and IFN-α. The up-regulation of expression of IL-1β, IL-8, and IL-10 and down-regulation in IFN-α was observed in both WT and CD163 SRCR5-edited PAMs infected with PRRSV compared to mock infected cells (Figure 8). However, there was no significant difference in the expression of IL-1β, IL-8, IL-10, and IFN-α between WT and CD163 SRCR5-edited PAMs regardless of whether cells were infected with PRRSV (Figure 8). These data suggest that CD163 SRCR5-edited PAMs present a cytokine response to PRRSV infection.

## Discussion

In this study, PEFs with targeted deletion of the SRCR5 region in CD163 were enriched using our previously developed dual fluorescence selection strategy (30). Using these enriched cells as donors for SCNT, we generated a high number of pigs harboring a 41-aa deletion of CD163 SRCR5. In a previous study, zygote injection of the same pair of sgRNAs resulted in only one out of four (25%) healthy piglets carrying the deletion of CD163 SRCR5 (29). More recently, a second study also performed zygote injection of paired sgRNAs and only obtained one out of 32 (3%) piglets carrying the expected deletion of CD163 exon 7 (34). Using our technique, we obtained a higher efficiency of deletion in Liang Guang Small Spotted pigs (3/8, 37.5%) and Large White pigs (9/13, 69%) (Tables S2, S3) through SCNT of sorted cells transfected with CRISPR/Cas9. Similar to our previous studies (35), we found that the newborn gene-edited pigs in general are weaker in viability compared to newborn WT piglets. This could be attributed to uncharacterized epigenetic changes, which may prevent the complete activation of the zygotic genome in SCNT embryos, resulting in a certain percentage of newborn gene-edited piglets with developmental abnormities (36). In addition, the workers in our cooperative pig farm have never nursed newborn gene-edited pigs, and this lack of experience contributed to the death of most piglets at an early stage, leaving behind a limited number of healthy founder pigs (Table 1).

PRRSV has been an epidemic for more than 20 years in China, where it was first isolated in 1995. Due to the high genetic diversity of PRRSV, several HP-PRRSV strains have emerged that lead to severe PRRS, which caused heavy economic losses in the swine industry worldwide. HP-PRRSV, including JXA1 and HuN4, a new PRRSV variant, caused nearly 20% mortality in pigs in 2006 (4). Since then, the HP-PRRSV-like strains have been identified as the dominant strains in China (37). To protect pigs from PRRSV infection, a modified live vaccine is commonly used throughout the swine industry worldwide. Due to the diversity of virus strains and the lack of cross-protection, current vaccines provide only limited protection (38). Moreover, no effective drugs exist for PRRSV treatment. Therefore, it is necessary to develop new anti-PRRSV strategies. The emerging gene-editing tool CRISPR/Cas9 has proven to be powerful for the precise genome modification of a variety of organisms (39). Therefore, we applied CRISPR/Cas9 to delete a short fragment in the SRCR5 domain of porcine CD163 to develop pig breeds resistant to PRRSV infection.

Previous studies have demonstrated that deletion of the entire SRCR5 domain of CD163 is sufficient to resist PRRSV infection while maintaining the biological function of CD163 (34). We further showed that a smaller and more precise modification of the CD163 SRCR5 domain was capable of conferring resistance to PRRSV infection. Deletion of the 41-aa fragment including the LBP region in SRCR5 gave rise to Large White pigs fully resistant to the infection of two type-2 PRRSV strains, JXA1 and MY. Our study thus strongly confirmed the essential role of LBP region of SRCR5 in its interaction with PRRSV. We speculate that deletion of other critical regions like loop 5–6 (23) in SRCR5 may also confer resistance to PRRSV infection. Our study provides a perspective on the generation of PRRSV resistant pigs through minimal modification of CD163 protein to maximally maintain its other biological functions.

Due to the acclimation of the dominant strain PRRSV in China, the viral strains used in this study seem to have become attenuated to mild virulence, and some pig breeds like the Large White may not show intense micro lesions in lung tissue and high fever above 40.5°C after PRRSV infection (Figures 4B, 5B). To demonstrate the full resistance of CD163 SRCR5-edited pigs to PRRSV infection, the challenge time of PRRSV strains JXA1 and MY was extended to 42 days (Figure 3). As CD163 SRCR5-partially deleted Liang Guang Small Spotted pigs were also generated in this study, it would be interesting to examine the difference in the intensity of micro lesions in lung tissue from this breed after viral challenge with the same two PRRSV strains in the future.

Interestingly, although CD163 SRCR5-edited PAMs were resistant to PRRSV infection and the expression of PRRSV ORF7 was undetectable at later infection time points, viral ORF7 mRNA was detectable at a very low level in SRCR5-edited cells at an early stage of infection (Figure 7F). The reason for this may be that the partial deletion of CD163 SRCR5 does not affect PRRSV attachment and internalization, but blocks virus uncoating in the early endosome, thus inhibiting viral genome release into cytoplasm. Subsequent virions are then transported to the late endosome and finally degraded in the lysosome (40). We do not have a proper explanation for the increased expression of CD163 mRNA over the course of infection in SRCR5-edited PAMs (Figure 7E). The underlying mechanisms need to be further investigated. In addition to its interaction with PRRSV, CD163 has many other functions, including the uptake of Hb-Hp and the regulation of inflammation by shedding soluble CD163 (sCD163) (41). Molecular characterization of PAMs showed that the small deletion in SRCR5 did not affect the normal expression of CD163 protein or the shedding of sCD163 in the culture medium of PAMs infected with PRRSV (Figure 7). These data imply that, unlike the CD163 knockout described in a previous study (25, 27), the small deletion in SRCR5 maintains the majority of the biological functions of CD163.

Surprisingly, although the small deletion of CD163 SRCR5 blocks the infection by PRRSV, we found that SRCR5-edited PAMs present a similar cytokine response to PRRSV challenge to the WT PAMs (Figure 8). The reason may be that, although virus uncoating and genome release are suppressed in CD163 SRCR5-partially deleted PAMs after PRRSV infection, the virion can adsorb and enter the cells during the early phase of infection (Figure 7F), which might induce cytokine expression in SRCR5-edited cells.

In conclusion, dual fluorescent selection was applied to enrich PEFs lacking a short region, which contains LBP in the SRCR5 region of CD163, and efficiently generate gene-edited pigs of two different breeds through SCNT. Precise deletion of the LBP region in CD163 SRCR5 confers Large White pigs full resistance to species 2 PRRSV infection, while maintaining the normal biological function of CD163. However, the response of CD163 SRCR5-edited pigs to other infectious diseases and their growth, reproduction, and other phenotypic features need to be further characterized to evaluate their potential breeding value and practical application in the future.

## Author Contributions

CG, HL, XiaohL, and ZH conceived and designed the study. CG, MW, ZZ, SH, and ZH performed the experiments, analyzed the data, and drafted the manuscript. CG, MW, ZZ, SH, HL, XiaofL, XS, TT, PY, JZ, LY, YCa, YCh, XiaohL, and ZH participate in pig management and sampling, read and approved the final manuscript. CG and ZH contributed to the interpretation of the data and took part in the critical revision of the manuscript.

### Conflict of Interest Statement

JZ and LY were employed by Guangdong YIHAO Food Co., Ltd. The remaining authors declare that the research was conducted in the absence of any commercial or financial relationships that could be construed as a potential conflict of interest.

## Supplementary Material

Figure S1 Dual fluorescence selection of PEFs transfected with paired sgRNAs. **(A)** The fluorescent images of PEFs 24 h after transfection of plasmids pX458-sgRNA10 and pX458R-sgRNA134. Bar = 100 μm. **(B)** Flow cytometry analysis of PEFs 48 h after transfection of plasmids pX458-sgRNA10 and pX458R-sgRNA134. 10.9% PEFs derived from Liang Guang Small Spotted pig and 4.7% PEFs derived from Large White pig expressing dual fluorescent proteins.

Figure S2 Genotypes of CD163 edited healthy piglets. **(A)** Sequence analysis of clone PCR products of healthy Liang Guang Small Spotted piglets. **(B)** Sequence analysis of clone PCR products of healthy Large White piglets.

Figure S3 Schematic overview of generation of CD163 edited pigs through SCNT of PEFs from dual fluorescence selection. PEFs were transfected with Cas9/sgRNA co-expression vectors pX458-sgRNA10 and pX458R-sgRNA134 which contain reporter EGFP and DsRed, respectively. PEFs simultaneously expressing EGFP and DsRed were collected through fluorescence activated cell sorting. Then the sorting cells were introduced into enucleated oocytes, fused and activated. When the reconstructed cloned embryos developing to 2-cell stage were transferred into the oviduct of the recipient sows. In this diagram, the edited cells were derived from Liang Small Spotted pig, and Large White sows were used as surrogates.
