# Supplementary figures and images for: Highly Efficient Generation of Pigs Harboring a Partial Deletion of the CD163 SRCR5 Domain, Which Are Fully Resistant to Porcine Reproductive and Respiratory Syndrome Virus 2 Infection
Source: Front Immunol. 2019 Aug 8;10:1846. doi: 10.3389/fimmu.2019.01846 (PMC6694839; doi:10.3389/fimmu.2019.01846)

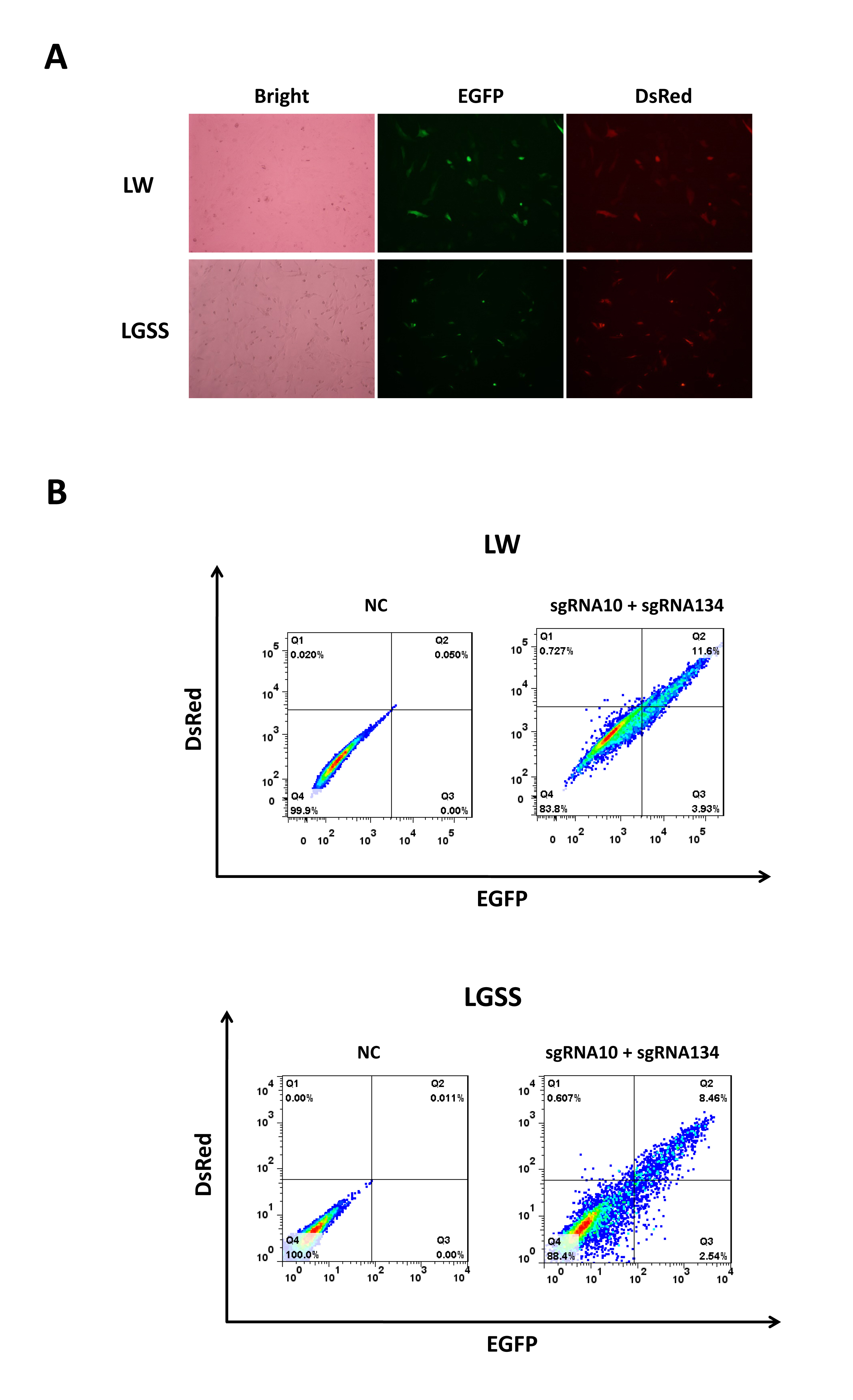

Supplement: Figure S1 — Dual fluorescence selection of PEFs transfected with paired sgRNAs. (A) The fluorescent images of PEFs 24 h after transfection of plasmids pX458-sgRNA10 and pX458R-sgRNA134. Bar = 100 μm. (B) Flow cytometry analysis of PEFs 48 h after transfection of plasmids pX458-sgRNA10 and pX458R-sgRNA134. 10.9% PEFs derived from Liang Guang Small Spotted pig and 4.7% PEFs derived from Large White pig expressing dual fluorescent proteins. [file Image_1.JPEG]

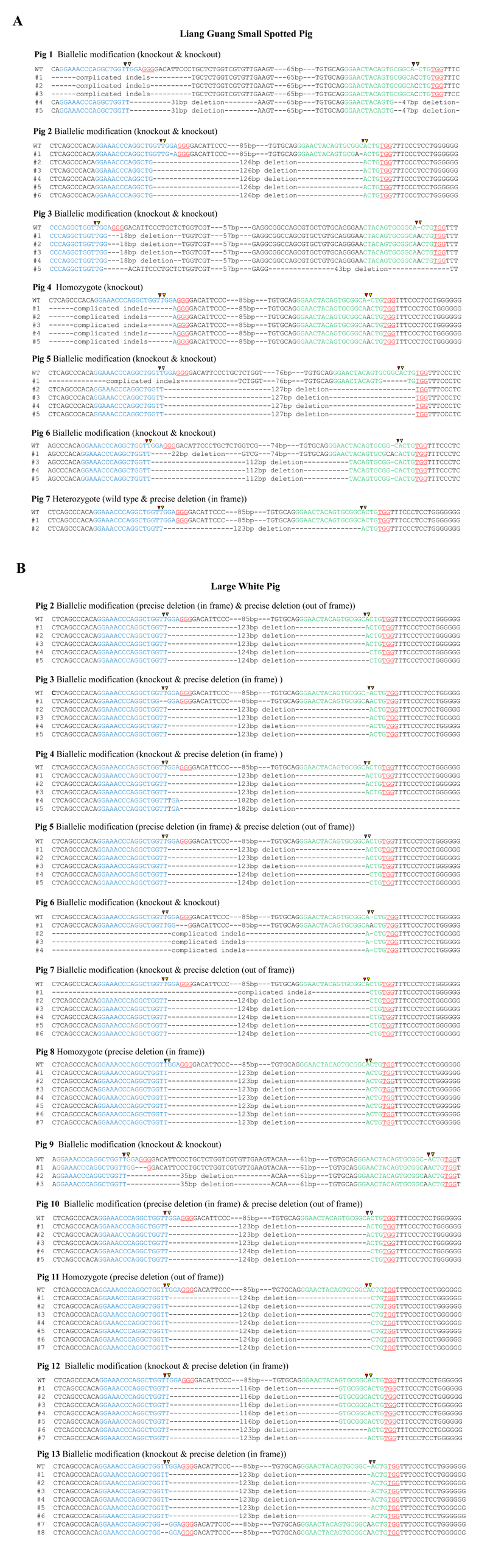

Supplement: Figure S2 — Genotypes of CD163 edited healthy piglets. (A) Sequence analysis of clone PCR products of healthy Liang Guang Small Spotted piglets. (B) Sequence analysis of clone PCR products of healthy Large White piglets. [file Image_2.TIF]

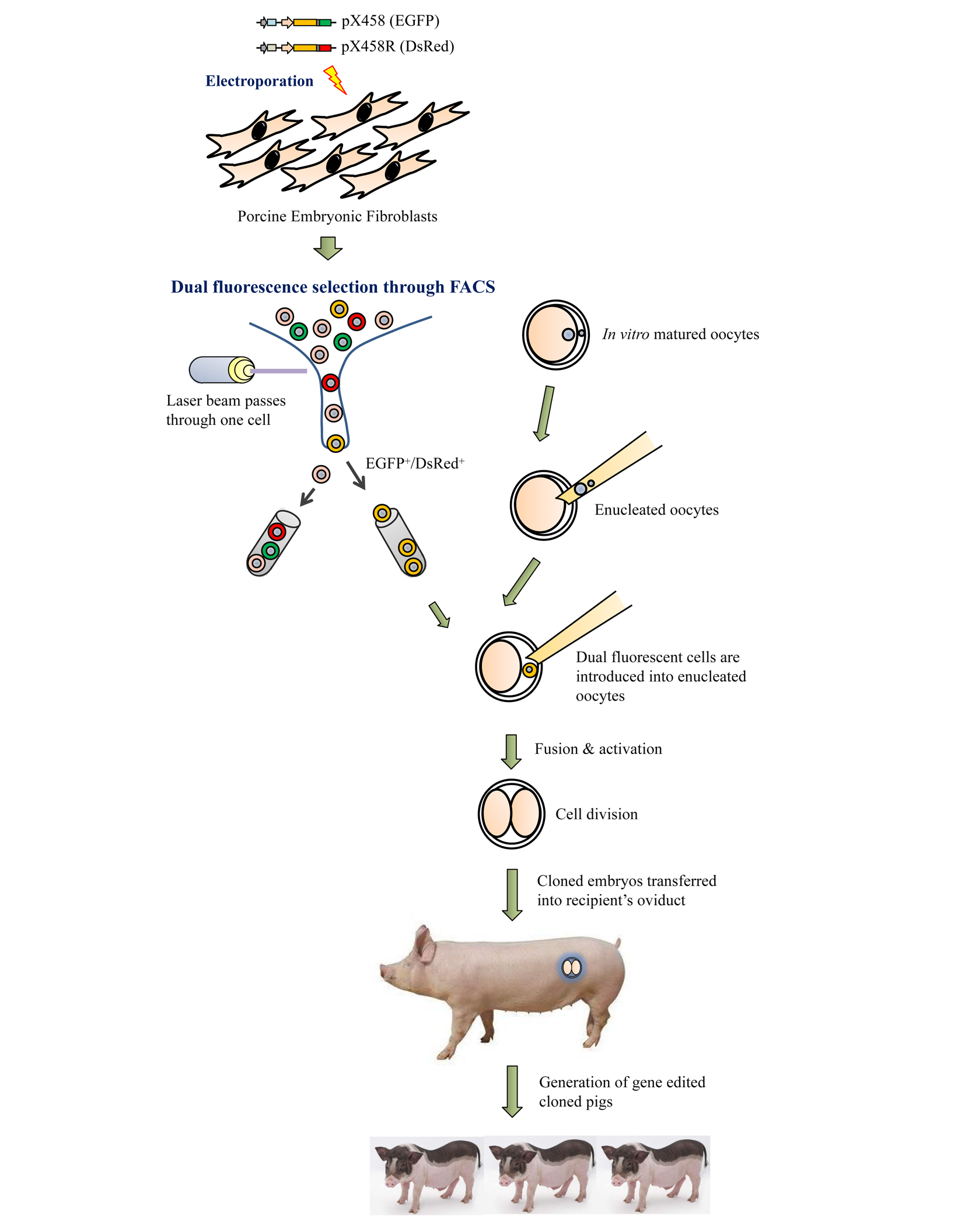

Supplement: Figure S3 — Schematic overview of generation of CD163 edited pigs through SCNT of PEFs from dual fluorescence selection. PEFs were transfected with Cas9/sgRNA co-expression vectors pX458-sgRNA10 and pX458R-sgRNA134 which contain reporter EGFP and DsRed, respectively. PEFs simultaneously expressing EGFP and DsRed were collected through fluorescence activated cell sorting. Then the sorting cells were introduced into enucleated oocytes, fused and activated. When the reconstructed cloned embryos developing to 2-cell stage were transferred into the oviduct of the recipient sows. In this diagram, the edited cells were derived from Liang Small Spotted pig, and Large White sows were used as surrogates. [file Image_3.TIF]
