# Supplementary material for: Highly Efficient Generation of Pigs Harboring a Partial Deletion of the CD163 SRCR5 Domain, Which Are Fully Resistant to Porcine Reproductive and Respiratory Syndrome Virus 2 Infection
Source: Front Immunol. 2019 Aug 8;10:1846. doi: 10.3389/fimmu.2019.01846 (PMC6694839; doi:10.3389/fimmu.2019.01846)
Supplement: Supplementary file 4 [file Data_Sheet_1.docx]

**Supplemental Table 1. Evaluation of the *in vitro* developmental competency of cloned edited embryos.**

| **Breed** | **No. Experiment** | **Embryo Type** | **No. Embryos** | **Cleaved Embryos (%)** | **Blastocysts (%)** |
| --- | --- | --- | --- | --- | --- |
| LGSS | 1 | NT | 125 | 84.85 % | 17.38 % |
|  | 2 | NT | 94 | 87.18 % | 16.89 % |
|  | 3 | NT | 180 | 91.66 % | 67.84 % |
|  | 4 | NT | 20 | 90.00 % | 50.00 % |
|  |  | PA | 118 | 91.53 % | 61.86 % |
| LW | 1 | NT | 168 | 91.07 % | 62.50 % |
|  | 2 | NT | 35 | 91.43 % | 48.57 % |
|  |  | PA | 101 | 99.01 % | 54.46 % |

NT: nuclear transfer. PA: parthenogenetic activation.

Cleaved Embryos (%) and Blastocysts (%) were evaluated separately on day 2 and day 7 after SCNT.

**Supplemental Table 2. Genotype and translational prediction for Liang Guang Small Spotted pigs with CD163 SRCR5 partial deletion including ligand-binding pocket.**

| **pig**  **ID** | **Type** | **Size of INDELs** | **Description** | **Protein translation** | **Premature**  **stop codon** |
| --- | --- | --- | --- | --- | --- |
| 1 | Biallelic | Complicated indels& 1 bp insertion;  78 bp deletion | One allele: unexpected complicated insertions and deletions & one base insertion on sgRNA 134 target site; Another allele: 31 bp deletion near sgRNA 10 target site, and 47 bp deletion near sgRNA 134 target site | KO  KO | ND  YES |
| 2 | Biallelic | 2 bp deletion & one substitution;  126 bp deletion | One allele: one base deletion on each target site& single base mutation(G>A) on sgRNA 134 target site; Another allele: partial deletion in exon 7 | KO  CD163^∆475-516^ | YES  NO |
| 3 | Biallelic | 18 bp deletion & 1 bp insertion;  49 bp deletion | One allele: partial deletion in exon 7 & one base insertion on sgRNA 134 target site; Another allele: 6 bp deletion near sgRNA 10 target site, and 43 bp deletion near sgRNA 134 target site | KO  KO | NO  YES |
| 4 | Homozygous | Complicated indels & 1 bp insertion | unexpected complicated insertions and deletions & one base insertion on sgRNA 134 target site | KO | ND |
| 5 | Biallelic | Complicated indels;  127 bp deletion | One allele: unexpected  complicated insertions and deletions; Another allele: partial deletion in exon 7 | KO  KO | ND  YES |
| 6 | Biallelic | 22 bp deletion & 1 bp insertion &one substitution;  112 bp deletion | One allele: partial deletion in exon 7 & one base insertion and single base mutation (G>C) near sgRNA 134 target site; Another allele: partial deletion in exon 7 | KO  KO | YES  YES |
| 7 | Heterozygous | Wild type;  123 bp deletion | One allele: wild type; Another allele: partial deletion in exon 7 | Wild type  CD163^∆476-516^ | NO  NO |
| 8 | Heterozygous | Wild type;  123 bp deletion | One allele: wild type; Another allele: partial deletion in exon 7 | Wild type  CD163^∆476-516^ | NO  NO |

ND: Not determined.

**Supplemental Table 3. Genotype and translational prediction for Large White pigs with CD163 SRCR5 partial deletion including ligand-binding pocket.**

| **Pig ID** | **Type** | **Size of INDELs** | **Description** | **Protein translation** | **Premature**  **stop codon** |
| --- | --- | --- | --- | --- | --- |
| 1^a^ | Homozygous | 123 bp deletion | Partial deletion in exon 7 | CD163^∆476-516^ | NO |
| 2^a^ | Biallelic | 123 bp deletion;  124 bp deletion | Partial deletion in exon 7 | CD163^∆476-516^  KO | NO  YES |
| 3^a^ | Biallelic | 2 bp deletion & 1 bp insertion;  123 bp deletion | One allele: 2 bp deletion on sgRNA 10 target site & one base insertion on sgRNA 134 target site; Another allele: partial deletion in exon 7 | KO  CD163^∆476-516^ | YES  NO |
| 4^b^ | Biallelic | 123 bp deletion  182 bp deletion& one substitution | One allele: partial deletion in exon 7; Another allele: partial deletion in exon 7 & base mutation (G>T) on sgRNA 10 target site | CD163^∆476-516^  KO | NO  YES |
| 5^b^ | Biallelic | 123 bp deletion;  124 bp deletion | Partial deletion in exon 7 | CD163^∆476-516^  KO | NO  YES |
| 6 | Biallelic | 3 bp deletion & 1bp insertion;  Complicated indels | One allele:3 bp deletion near sgRNA 10 target site & one base insertion on sgRNA 134 target site; Another allele: unexpected complicated insertions and deletions | KO  KO | NO  ND |
| 7 | Biallelic | 124 bp deletion;  Complicated indels | One allele: partial deletion in exon 7; Another allele: unexpected complicated insertions and deletions | KO  KO | YES  ND |
| 8^b^ | Homozygous | 123 bp deletion | Partial deletion in exon 7 | CD163^∆476-516^ | NO |
| 9 | Biallelic | 3 bp deletion & 1 bp insertion;35 bp deletion & 1 bp insertion | One allele: 3 bp deletion near sgRNA 10 target site & one base insertion on sgRNA 134 target site; Another allele: Partial deletion in exon 7 & one base insertion on sgRNA 134 target site | KO  KO | NO  YES |
| 10^b^ | Biallelic | 123 bp deletion;  124 bp deletion | Partial deletion in exon 7 | CD163^∆476-516^  KO | NO  YES |
| 11 | Homozygous | 124 bp deletion | Partial deletion in exon 7 | KO | YES |
| 12^a^ | Biallelic | 116 bp deletion  123 bp deletion | Partial deletion in exon 7 | KO  CD163^∆476-516^ | NO  NO |
| 13 | Biallelic | 123 bp deletion;  2 bp deletion & 1 bp insertion | One allele: partial deletion in exon 7; Another allele: 2 bp deletion on sgRNA 10 target site & one base insertion on sgRNA 134 target site | CD163^∆476-516^  KO | NO  YES |

a: For HP-PRRSV JXA1 strain challenge, b: For HP-PRRSV MY strain challenge, ND: Not determined.

**Supplemental Table 4. List of primers for qRT-PCR.**

| Primer^a^ | Sequence (5'-3')^b^ |
| --- | --- |
| ORF7-F | AAAACCAGTCCAGAGGCAAG |
| ORF7-R | CGGATCAGACGCACAGTATG |
| HPRT1-F | TGGAAAGAATGTCTTGATTGTTGAAG |
| HPRT1-R | ATCTTTGGATTATGCTGCTTGACC |
| CD163-F | ATTCATCATCCTCGGACCCAT |
| CD163-R | CCCAGCACAACGACCACCT |
| IL-1β-F | CCCAAAAGTTACCCGAAGAGG |
| IL-1β-R | TCTGCTTGAGAGGTGCTGATG |
| IL-8-F | AGTTTTCCTGCTTTCTGCAGCT |
| IL-8-R | TGGCATCGAAGTTCTGCACT |
| IL-10-F | TGAGAACAGCTGCATCCACTTC |
| IL-10-R | TCTGGTCCTTCGTTTGAAAGAAA |
| IFN-α-F | TCCAGCTCTTCAGCACAGAG |
| IFN-α-R | AGCTGCTGATCCAGTCCAGT |

a: F, forward primer; R, reverse primer.

b: Porcine sequences and PRRSV gene sequences were downloaded from GenBank.
